# A case of indirect inguinal bladder hernia treated with laparoscopic transabdominal preperitoneal repair with high peritoneal incisional approach

**DOI:** 10.1186/s40792-024-01860-7

**Published:** 2024-03-20

**Authors:** Hitomi Zotani, Tetsu Yamamoto, Ryoji Hyakudomi, Kiyoe Takai, Takahito Taniura, Kazunari Ishitobi, Noriyuki Hirahara, Yoshitsugu Tajima, Masaaki Hidaka

**Affiliations:** Department of Digestive and General Surgery, Shimane University Faculty of Medicine, 89-1 Enya, Izumo, Shimane 693-8501 Japan

**Keywords:** Groin hernia, Inguinal hernia, Bladder hernia, Laparoscopic surgery, TAPP, High peritoneal incision approach (HPIA)

## Abstract

**Background:**

Inguinal herniation of the urinary bladder is uncommon and those descending into the scrotum are even rarer. Although open anterior repair has been used for inguinal bladder hernia, the efficacy of laparoscopic herniorrhaphy has been reported in recent years.

**Case presentation:**

A 63-year-old man presented with an irreducible right groin and scrotal bulge associated with voiding difficulty. Abdominal ultrasonography showed a dislocation of the urinary bladder descending into the right scrotum. Abdominal CT imaging revealed that a part of the bladder and small intestine was herniating into the scrotum through the internal inguinal ring and running laterally to the inferior epigastric artery. Under the diagnosis of indirect inguinal bladder hernia, the patient underwent trans-abdominal preperitoneal hernia repair (TAPP). The bladder herniated into the scrotum through the internal inguinal ring was replaced to the original position. Then the myopectineal orifice was exposed and covered with polypropylene mesh, where a horizontal peritoneal incision 4 cm above the hernia orifice, i.e., the high peritoneal incision approach (HPIA), allowed an easy peeling of the peritoneum and hernia sac. The patient’s postoperative course was uneventful and the voiding difficulty resolved. The patient continued to do well without recurrence at 20 months after surgery.

**Conclusion:**

Preoperative evaluation with abdominal ultrasonography and CT scan allowed a precise diagnosis of a groin hernia with voiding difficulty. TAPP with HPIA was useful in the treatment of inguinal bladder hernia because this technique facilitated a quick confirmation of the hernia contents, secure dissection of the whole protruded bladder, and adequate replacement of the bladder to the original position without any injury.

## Background

Inguinal hernia is one of the most common diseases and it usually contains the omentum and small intestine. Meanwhile, inguinal herniation of the urinary bladder is rare, first described by Felix Platter and Dominic Sala in the sixteenth century [1], with an incidence of 1–4% of inguinal hernias [1–4], while massive inguinoscrotal bladder hernia reaching to the scrotum is even rarer [5]. Most inguinal bladder hernias are the direct type and are divided into three types: paraperitoneal type, extraperitoneal type, and intraperitoneal type [6]. Although anterior herniorrhaphy has been the gold standard approach for inguinal bladder hernia, recent reports have described the efficacy of laparoscopic herniorrhaphy, such as total extraperitoneal (TEP) hernia repair and transabdominal preperitoneal (TAPP) hernia repair [7]. Additionally, TAPP is better at avoiding bladder injury because it can identify the hernial sac at the beginning of the laparoscopy, so there is no misidentification of a protruded bladder as the hernial sac [8].

When performing the TAPP repair, we usually use a high peritoneal incision approach (HPIA), in which a horizontal peritoneal incision is made 4 cm above the hernia orifice, then the peritoneum is peeled. This procedure facilitates an easier and more suitable dissection of the hernia sac by tracing a dissectible layer between the peritoneum and preperitoneal fascia, even in cases with inflammatory adhesions. We herein report a rare case with a massive, indirect inguinoscrotal hernia of the urinary bladder treated with TAPP repair with HIPA.

## Case presentation

A 63-year-old man with complaints of voiding difficulty and a right inguinal bulge for 6 years was referred to our hospital.

## Physical examination

The right inguinal bulge with swelling of the right scrotum was apparent and irreducible. There was no pain or tenderness.

## Laboratory data

Laboratory data were within normal ranges, including white blood cell count and C-reactive protein.

## Abdominal ultrasonography

Abdominal ultrasound examination showed a dislocation of the urinary bladder descending into the right scrotum.

## Abdominal CT scan

**Fig. 1:**
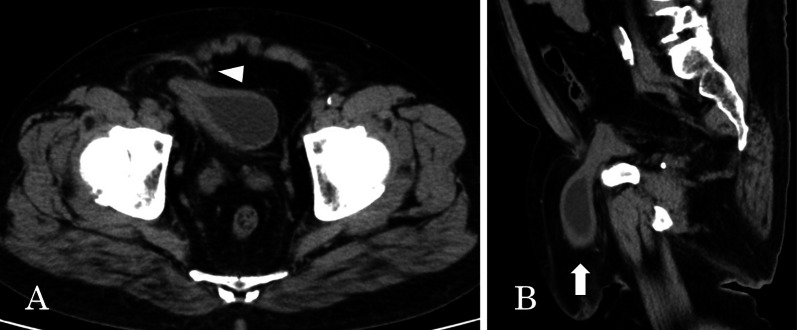
CT scan findings. Abdominal and pelvic CT scans show the right indirect inguinal bladder hernia. **A** A part of the bladder protruded outside the inferior epigastric vessels (white arrowhead). **B** The protruded bladder (white arrow) containing urine ran into the scrotum

Abdominal and pelvic CT imaging studies revealed that a part of the bladder and small intestine was running laterally to the inferior epigastric artery and herniating into the scrotum through the internal inguinal ring, indicating an indirect inguinal bladder hernia (Fig. 1).

## Cystoscopy

Although a cystoscopy was performed to investigate the cause of the voiding difficulty, there were no abnormal findings both in the urethra or urinary bladder.

The patient was thus diagnosed with an incarcerated right indirect inguinal bladder hernia and underwent an elective laparoscopic TAPP repair with HPIA.

## Operation findings

The patient was placed in the supine position under general anesthesia combined with a transversus abdominis plane block. A 5-mm trocar for the camera (flexible laparoscope) was placed slightly above the umbilicus by optical access [9]. After creating a pneumoperitoneum with carbon dioxide at an intra-abdominal pressure of 10 mmHg, two additional 5-mm trocars were placed bilaterally at the levels of the umbilicus.

**Fig. 2:**
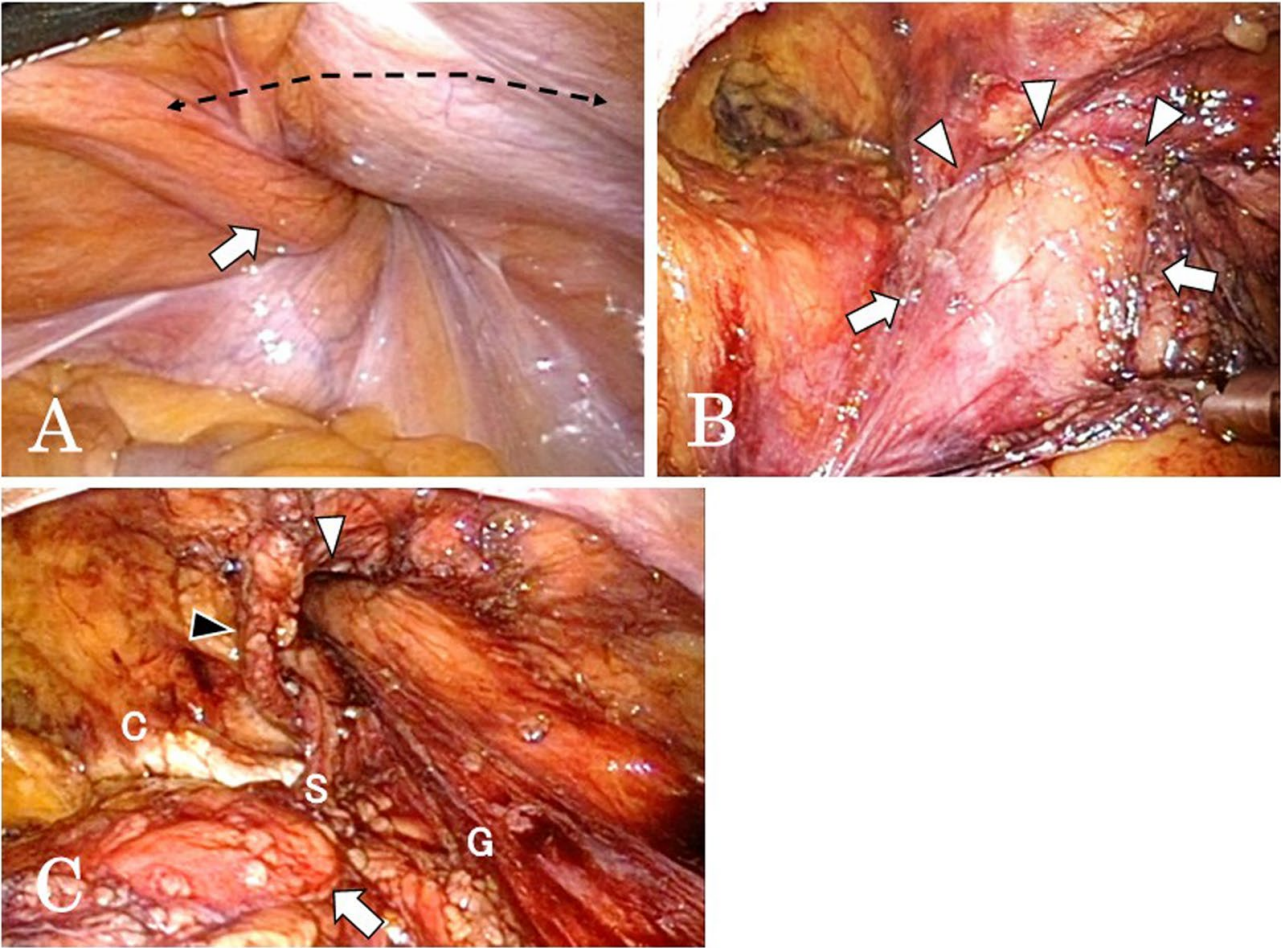
Surgical findings. **A** A high peritoneal incision approach (HPIA) for inguinal hernia (dotted line). The right inguinal bladder hernia of paraperitoneal type was recognized (arrow). **B** The bladder (arrows) was incarcerated through the right internal inguinal ring (arrowheads). **C** The protruded bladder was returned to the abdominal cavity (arrow). The hernial orifice (white arrowhead) was placed outside the inferior epigastric vessels (black arrowhead) and MPO was exposed. **C** Cooper ligament. *S* spermatic cord. *G* Gonadal vessels

The right indirect inguinal hernia with a protruded urinary bladder was found. It was difficult to pull through the protruded bladder because the bladder hernia was the paraperitoneal type. At first, a horizontal peritoneal incision was made 4 cm above the hernial orifice, from the anterior superior iliac spine to the plica umbilicalis medialis, i.e., the HPIA, and dissection of the preperitoneal space was performed. After the hernia sac was dissected around the hernial orifice, it was confirmed that part of the bladder protruded into the scrotum through the hernial orifice (Fig. 2). Adhesiolysis was necessary and time-consuming because of the firm adhesion between the protruding bladder and the inguinal canal. After that the protruded bladder was returned to the normal position, followed by exposure of the myopectineal orifice (MPO). The hernial orifice was wide, 4 cm in diameter, and a surgical mesh for the hernia, 15 × 10 cm lightweight polypropylene mesh (TiLENE® mesh extra light, PFM Medical, Inc., Carlsbad, CA), was placed covering the MPO and tacked with an absorbable tacker (AbsorbaTack™, Medtronic Japan Co., Ltd., Tokyo, Japan). The peritoneum was closed by a continuous suture. The operative time was 165 min with minimal blood loss.

The patient’s postoperative course was uneventful and the voiding difficulty was completely resolved. The patient was discharged 2 days after the operation and continued to do well without recurrence at 20 months after surgery.

## Discussion

The urinary bladder hernia is defined as a state of sliding a part or whole bladder wall to the inguinal canal, with an incidence of 1–4% of all adult inguinal hernias [1–4]. The bladder hernia is more common in males in their 50 s than females and usually presents a groin bulge associated with voiding difficulty, double voiding, or frequent urination. The risk factors of bladder hernia are suggested to be obesity, weakening of the bladder tone and abdominal pelvic wall, and increasing intravesical pressure [10]. Soloway et al. [6] classified bladder hernias into three categories paraperitoneal type, extraperitoneal type, and intraperitoneal type. The paraperitoneal type is the most common type and is defined as the extraperitoneal portion of the bladder is involved and lies along the inner wall of the sac. Extraperitoneal type is siding only the bladder wall to the inguinal canal. Intraperitoneal type, the second most common type, is defined as the bladder entering hernia sac, thus completely covered by the peritoneum [6]. In our case, the bladder hernia was categorized as the paraperitoneal type. The patient complained of voiding difficulty in urination associated with bladder hernia because his symptoms improved completely after surgery.

Preoperative imaging studies such as abdominal ultrasonography and CT scan are useful for the diagnosis of inguinal urinary bladder hernia [11]. Detailed evaluation with cystography or cystoscopy is also needed because a high incidence of urological malignancies (11.2%) is reported in patients with bladder hernia [12]. Unfortunately, preoperative definitive diagnosis of inguinal bladder hernia is reported to be less than 7%, and most cases are diagnosed at surgery [12]. In addition, the risk of bladder injury during surgery for inguinal bladder hernia reaches 12%, especially in cases with chronic enlarged prostate or pericystitis with firm adhesions [12, 13]. The resection of the bladder is recommended in cases with bladder tumors or necrosis, or diverticulum existing on the bladder [14, 15]. In this case, ultrasonography confirmed the bladder protruded into the inguinal canal when the bladder filled with urine, and cystoscopy showed no tumor or mucosal abnormality in the bladder. An abdominal CT scan identified the positional relationship between the inferior epigastric vessels and the protruded bladder and ruled out the presence of a bladder tumor. A precise preoperative imaging study is thus necessary for categorizing the bladder hernia type accurately, making herniorrhaphy safer, and avoiding intraoperative bladder injury.

Most patients with an inguinal bladder hernia have received open, tension-free repair, but recent laparoscopic herniorrhaphy seems to be more useful because this approach can confirm the type of bladder hernia and adequately return the protruded bladder to the normal position. There are two procedures for laparoscopic herniorrhaphy such as TAPP and TEP. Since we can diagnose the bladder hernia by precise imaging and classify it into three types preoperatively, we suggest that these procedures be selected according to the type of bladder hernia. For the paraperitoneal type, TAPP is useful because the contents of the hernia can be detected at the beginning of the surgery and injury to the bladder or bowel can be avoided. Next, TEP seems to be preferable for the extraperitoneal type because the prolapsed bladder can be easily detected during dissection and the bladder can be reduced to its normal position. Finally, for the intraparietal type, both procedures can be performed safely. Surgeons should use their experience to decide which procedure to use. Meanwhile, there are concerns about intraoperative bladder injury during laparoscopic surgery due to technical difficulties [16, 17]. Therefore, further studies are needed to clarify the superiority of laparoscopic herniorrhaphy for inguinal bladder hernia. In the present case, we utilized the HPIA technique which starts the peritoneal incision 4 cm above the hernial orifice and dissects at the dissectible layer to the hernia sac. This approach facilitated an easy and safe dissection around the protruded bladder in a cranial-to-caudal manner, leading to an accurate recognition of the whole protruded bladder and an avoidance of intraoperative bladder injury. In addition, in the case of bladder hernia observed during TAPP, the circular incision technique, which incises directly at the hernia orifice, may cause injury to the bladder. Therefore, HIPA TAPP will be advantageous in avoiding such intraoperative adverse events.

## Conclusions

Preoperative detailed evaluation of the urinary bladder with abdominal ultrasonography, CT scan, and cystography/cystoscopy is important in the management of inguinal bladder hernia. TAPP with HPIA is useful for inguinal bladder hernias because this technique enables us to overlook the entire bladder and replace the protruded bladder to the original position after a secure dissection, without injury to the bladder.

## Data Availability

No data analysis was performed in this article, so there is no data to submit.
